# Introgression of Two Broad-Spectrum Late Blight Resistance Genes, *Rpi-Blb1* and *Rpi-Blb3*, From *Solanum bulbocastanum* Dun Plus Race-Specific *R* Genes Into Potato Pre-breeding Lines

**DOI:** 10.3389/fpls.2020.00699

**Published:** 2020-06-03

**Authors:** Elena Rakosy-Tican, Ramona Thieme, Janine König, Marion Nachtigall, Thilo Hammann, Tunde-Eva Denes, Klaudia Kruppa, Márta Molnár-Láng

**Affiliations:** ^1^Plant Genetic Engineering Group, Department of Molecular Biology and Biotechnology, Babeş-Bolyai University, Cluj-Napoca, Romania; ^2^Federal Research Centre for Cultivated Plants, Institute for Breeding Research on Agricultural Crops, Julius Kühn-Institut, Quedlinburg, Germany; ^3^Federal Research Centre for Cultivated Plants, Institute for Breeding Research on Horticultural Crops, Julius Kühn-Institut, Quedlinburg, Germany; ^4^Biological Research Centre, Jibou, Romania; ^5^Centre for Agricultural Research, Hungarian Academy of Sciences, Martonvásár, Hungary

**Keywords:** effectors, detached leaf assay, introgression breeding, late blight field resistance, gene specific markers, processing qualities, tuber yield

## Abstract

There is a wealth of resistance genes in the Mexican wild relative of cultivated *Solanum*, but very few of these species are sexually compatible with cultivated *Solanum tuberosum.* The most devastating disease of potato is late blight caused by the oomycete *Phytophthora infestans* (*Pi*). The wild hexaploid species *S. demissum*, which it is able to cross with potato, was used to transfer eleven race-specific genes by introgressive hybridization that were subsequently widely used in potato breeding. However, there are now more virulent races of *Pi* that can overcome all of these genes. The most sustainable strategy for protecting potatoes from late blight is to pyramid or stack broad-spectrum resistance genes into the cultivars. Recently four broad-spectrum genes (*Rpi*) conferring resistance to *Pi* were identified and cloned from the sexually incompatible species *S. bulbocastanum*: *Rpi-blb1* (RB), *Rpi-blb*2, *Rpi-blb*3, and *Rpi-bt1*. For this research, a resistant *S. bulbocastanum* accession was selected carrying the genes *Rpi-blb1* and *Rpi-blb3* together with race-specific *R3a* and *R3b* genes. This accession was previously used to produce a large number of somatic hybrids (SHs) with five commercial potato cultivars using protoplast electrofusion. In this study, three SHs with cv. ‘Delikat’ were selected and backcross generations (i.e., BC_1_ and BC_2_) were obtained using cvs. ‘Baltica’, ‘Quarta’, ‘Romanze’, and ‘Sarpo Mira’. Their assessment using gene-specific markers demonstrates that these genes are present in the SHs and their BC progenies. We identified plants carrying all four genes that were resistant to foliage blight in greenhouse and field trials. Functionality of the genes was shown by using agro-infiltration with the effectors of corresponding *Avr* genes. For a number of hybrids and BC clones yield and tuber number were not significantly different from that of the parent cultivar ‘Delikat’ in field trials. The evaluation of agronomic traits of selected BC_2_ clones and of their processing qualities revealed valuable material for breeding late blight durable resistant potato. We show that the combination of somatic hybridization with the additional use of gene specific markers and corresponding *Avr* effectors is an efficient approach for the successful identification and introgression of late blight resistance genes into the potato gene pool.

## Introduction

Late blight caused by *Phytophthora infestans* (Mont.) de Bary (*Pi*) is one of the most damaging diseases of potatoes as it does not only affect the above ground parts of the plants but also the tubers (Colton et al., 2006). Although the *Pi* genome has been sequenced (Haas et al., 2009), a deep understanding of the complex interaction and co-evolution of this pathogen and its host is lacking (Kamoun and Smart, 2005; Haverkort et al., 2009; Aguilera-Galvez et al., 2018). However, its potential in causing an epidemic coupled with its great adaptability led to many outbreaks and destructions of potato harvests during the course of human history (Vreugdenhil, 2007; Chakrabarti et al., 2017). Even today, the cost of control and lost production are estimated six billion euros per year, with half of this cost incurred in Europe (Haverkort et al., 2016). Current disease control relies on ten up to 16 fungicide applications per season (Hanson et al., 2007; Haverkort et al., 2009). Fungicides are not only costly but they pollute the environment and affect human health (Paro et al., 2012). Moreover, late blight resistance introduced into potato cultivars by classical breeding, based on eleven race-specific resistance genes from *S. demissum* was quickly overcome by new races of *Pi*.

*Solanum bulbocastanum* (*blb*), a wild diploid species of Mexican potato (2*n* = 2*x* = 24), is highly resistant to all known races of *Pi*, even when exposed to high concentrations of its spores. This species is a typical example of a 1EBN (endosperm balance number) species and hence cannot be conventionally crossed with cultivated potato (Johnston et al., 1980). To date, four of its Nucleotide Binding Sites–Leucine Rich Repeats (NBS-LRR) resistance genes have been identified and cloned: *Rpi-blb1* (Van der Vossen et al., 2003), also known as *RB* and *Rpi-bt1–*both loci located on chromosome VIII (Song et al., 2003; Oosumi et al., 2009), *Rpi-blb2–*on chromosome VI (Van der Vossen et al., 2005) and *Rpi-blb3* on chromosome IV (Lokossou et al., 2009). Nevertheless, it is likely there are other factors involved in its late blight resistance (Hein et al., 2009; Lokossou et al., 2010; Rietman, 2011). Horizontal or durable resistance is thought to be polygenic and hence a quantitative trait, with complex pathogen and host interaction mechanisms that are still poorly understood (Colton et al., 2006). For instance, it was suggested that the pathogenesis related protein StPRp27 contributes to a race-nonspecific resistance against *Pi* by inhibiting the development of the disease and could potentially be used in breeding for durable resistance to late blight (Shi et al., 2012).

Many of the *Rpi* genes are located in meta-QTLs, like *Rpi-blb2* on chromosome VI and *Rpi-blb3* on chromosome IV (Danan et al., 2011). The *Rpi* genes from *S*. *bulbocastanum* are considered to be broad spectrum resistance genes, as already shown for *Rpi-blb1* (Song et al., 2003), *Rpi-blb2* (Van der Vossen et al., 2005; Orbegozo et al., 2016), and *Rpi-blb3* (Zhu et al., 2012). The race-specific genes used in this study, originally identified in *S. demissum*, are *R3a* and *R3b*, which are members of the *R3* complex locus on chromosome XI. The *R2* gene recognizes the same effector as *Rpi-blb3*, coded by *Avr2* gene (Aguilera-Galvez et al., 2018). Huang et al. (2004) showed that the *R3* locus harbours two functionally distinct genes: *R3a* and *R3b*. The *R3a* gene is classified in the fast evolving class I (Huang et al., 2005). The *R3* locus in potato is an example of natural stacking of resistance genes. It contains the two closely linked *R3a* and *R3b* genes, which are 0.4 cM apart, with distinctly different resistances to particular types of *Pi*, *R3a* recognizes *Avr3a*, while *R3b* despite its sequence being similar to that of *R3a*, recognizes another effector encoded by the *Avr3b* gene (Li et al., 2011).

It has been shown that by combining broad-spectrum resistance genes by means of genetic engineering (cisgenesis), two or more gene stacks can confer durable late blight resistance in the field (Zhu et al., 2012; Haesaert et al., 2015). Unfortunately, genetic engineering, which uses cloned resistance genes from wild species of potato, is still not considered as non-genetically modified organism (GMO) and its use is restricted in Europe (directive 2001/18/EC), and globally by skeptical consumers. Despite considerable progress in the genetic analysis of quantitative resistance to late blight, under long day conditions, based on molecular markers (Gebhardt and Valkonen, 2001; Simko, 2002), breeders have made little progress in breeding resistant cultivars using marker-assisted selection (MAS). The major drawbacks are the tetrasomic inheritance of potato and the strong linkage between foliage resistance and late maturity (Bradshaw et al., 2004). On the other hand, for more than 100 years there was little progress in obtaining resistant cultivars by introducing single genes from *S. demissum* and conventional breeding using bridging species is very time consuming. This was the case for the commercial potato varieties ‘Bionica’ and ‘Toluca’ (Haverkort et al., 2009) in which the single broad-spectrum resistance gene *Rpi-blb2* was incorporated. It took more than 46 years of conventional breeding in Netherlands that started at the end of the fifties and used the late blight resistant *blb* as the source of the resistance and *S. acaule* as the bridging species (Hermsen and Ramanna, 1973).

Finding methods of deploying major resistance genes to *Pi* remains an important goal of potato breeding. Currently breeders isolate variants of *R* genes and deploy them in pyramids or stacks. It is expected that this will result in broad-spectrum recognition of *Pi* isolates and might provide a more durable resistance in the field (Jo et al., 2016). Recent screening of germplasm has revealed *Rpi* genes in wild species of *Solanum*, particularly broad-spectrum resistance genes in *blb* species (Wang et al., 2008, 2013; Hein et al., 2009). The current challenge is to select, judiciously combine and deploy sets of different resistance genes that confer durable late blight resistance on modern potato cultivars.

Previously, a large number of somatic hybrids (SHs) between potato cultivars and *S. bulbocastanum* were regenerated and analyzed for hybridity, ploidy and fertility (Rakosy-Tican et al., 2015). The goal of this study was to transfer broad-spectrum resistance genes *Rpi-blb1* and *Rpi-blb3* from *S*. *bulbocastanum* together with race-specific *R* genes (*R3a* and *R3b*), into the potato gene pool by somatic hybridization. A functional profiling of resistance genes by effectoromics, phenotypic expression of resistance in detached leaf assay and quantitative resistance assessment in the field were used to evaluate the resistance of the somatic hybrids (SHs) and derived back-cross progenies (BC_1_ and BC_2_). We tested the hypothesis if the four resistance genes introgressed by somatic hybridization present in the back-cross progenies confer resistance that can be confirmed in a greenhouse and field trials. Thus, the pre-breeding lines produced will be a valuable material for breeding durable late blight resistance potato cultivars.

## Materials and Methods

### Plant Material

The morphology and nuclear genetic constitution of the SHs and backcross (BC) progenies, BC_1_ and BC_2_, between five commercial potato cultivars (‘Baltica’, ‘Delikat’, ‘Quarta’, ‘Romanze’, and ‘Sarpo Mira’) and *S. bulbocastanum* accession GLKS–31741 (*blb*41) (Groß Lüsewitz Potato Collections (GLKS) of the IPK Genebank, Leibniz-Institute of Plant Genetics and Crop Plant Research, Germany) have been analyzed (Rakosy-Tican et al., 2015). Only fertile SHs were chosen for further resistance assays. These included three hybrids with potato cv. ‘Delikat’ carrying all four resistance genes and their derived BC clones (see Tables 1–3).

**TABLE 1:** Assessment of the somatic hybrid (SH) 82/4, between potato cv. ‘Delikat’ and *Solanum bulbocastanum* (*blb*41) and derived back-crossed progenies BC_1_ and BC_2_ (the cultivar used for crossing is indicated), for the presence of the resistance genes as revealed by gene specific markers, functional profiling of *Avr* genes by agro-infiltration, resistance to late blight in a detached leaf assay (DLA) and in the field (Δ-rAUDPC); standard varieties ‘Adretta’ (susceptible) and ‘Sarpo Mira’ (resistant to late blight) were used; the year of the field assays is given as a subscript to each value of Δ-rAUDPC; from each genotype three (*two) plants and three leaflets were infiltrated, hypersensitive reaction is indicated by HR and lack of reaction by 0: the numbers in brackets indicate the number of repetitions with the same reaction.

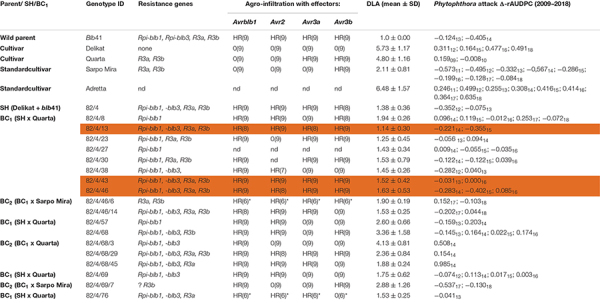

*Progenies with four stacked resistance genes, showing HR reaction and improved resistance to *Pi* in DLA and the field are highlighted in red; nd-not determined.*

**TABLE 2:** Assessment of the somatic hybrid (SH) 83/9, between potato cv. ‘Delikat’ and *Solanum bulbocastanum* (*blb*41) and derived back-crossed progenies BC_1_ (the cultivar used for crossing is indicated), for the presence of the resistance genes as revealed by gene specific markers, functional profiling of *Avr* genes by agro-infiltration, resistance to late blight in a detached leaf assay (DLA) and in the field (Δ-rAUDPC); standard varieties ‘Adretta’ (susceptible) and ‘Sarpo Mira’ (resistant to late blight) were used; the year of the field assays is given as a subscript to each value of Δ-rAUDPC; from each genotype three plants and three leaflets were infiltrated, hypersensitive reaction is indicated by HR and lack of reaction by 0: the numbers in brackets indicate the number of repetitions with the same reaction.

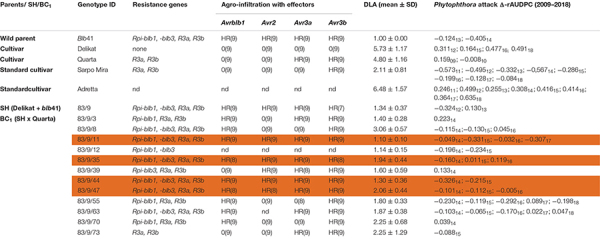

*Progenies with four stacked resistance genes, showing HR reaction and improved resistance to *Pi* in DLA and the field are highlighted in red; nd-not determined.*

**TABLE 3:** Assessment of the somatic hybrid (SH) 95/1, between potato cv. ‘Delikat’ and *Solanum bulbocastanum* (*blb*41) and derived back-crossed progenies BC_1_ and BC_2_ (the cultivar used for crossing is indicated), for the presence of the resistance genes as revealed by gene specific markers, functional profiling of *Avr* genes by agro-infiltration, resistance to late blight in a detached leaf assay (DLA) and in the field (Δ-rAUDPC); standard varieties ‘Adretta’ (susceptible) and ‘Sarpo Mira’ (resistant to late blight) were used; the year of the field assays is given as a subscript to each value of Δ-rAUDPC; from each genotype three plants and three leaflets were infiltrated, hypersensitive reaction is indicated by HR and lack of reaction by 0: the numbers in brackets indicate the number of repetitions with the same reaction.

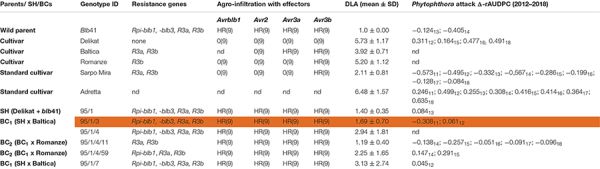

*BC_1_ clone with four stacked resistance genes, showing HR reaction and improved resistance to *Pi* in DLA and the field are highlighted in red; nd-not determined.*

### Assessment of Resistance Genes

#### Screening for Rpi-blb Resistance Genes

Fusion parents, SHs and their BC progenies were screened for the resistance genes *Rpi-blb1* using specific markers (Wang et al., 2008), *Rpi-blb3* (Lokossou et al., 2009; Zhu et al., 2012), *R3a* (Huang et al., 2005), and *R3b* (Rietman, 2011). Amplifications were carried out in a 20 μL reaction mixture containing 1.5 mM MgCl_2_, 200 μM of each dNTP, 0.5 μM primer, approximately 10 ng template DNA and 0.5 Unit InviTaq DNA Polymerase (Invitek). PCR was conducted using a standard set of conditions on a MJ Thermal cycler (PTC 200): initial denaturizing at 95°C for 5 min, followed by 35 cycles of 95°C for 30 s, T_*annealing*_ for 45 s, 72°C for 45 s and a final extension step of 72°C for 5 min. Amplification products were size-separated on a 1.5% agarose gel stained with ethidium bromide and visualized using a UV transilluminator. The expected sizes of amplified fragments were 821 bp for *Rpi-blb1*–marker Blb1 F/R (Wang et al., 2008), and 757 bp for *Rpi-blb3–*marker Blb3 F/R (Lokossou et al., 2009). For the race-specific genes previously characterized in *S. demissum*, the sizes of amplified fragments were 982 bp for *R3a* (Huang et al., 2005) and 378 bp for *R3b* (Rietman, 2011).

#### Agro-Infiltration

Agro-infiltration was done using a culture of the *A. tumefaciens* strain AGL1 + pVirg transformed with a pK7WG2 plasmid containing the avirulence factors for: *Rpi-blb1* (*Avrblb1*), *Rpi-blb3* (*Avr2* - recognized also by *R2*), *R3a* and *R3b* (*Avr3a* and *Avr3b*), obtained from the University of Wageningen (V. Vleeshouwers). The PITG codes are: *Avr2* = PITG_22870; *Avr3a* = PITG_14371; *Avr3b* = PITG_18215; and *Avrblb1* = PITG_21388.

Cultivation and infiltration was performed as described by Du et al. (2014). The macroscopic response was scored 4 days after infiltration (dpi), when the dead cells (hypersensitive reaction, HR) were visible. As a negative control, the pK7WG2 empty vector was used (Vleeshouwers et al., 2008, 2011), which had the advantage that the cells killed by the infiltration (background necrosis) can be detected and hence, were not scored as a false positive. The parents were used as positive controls. The agro-infiltration and evaluation was done on three leaflets of one plant and the final appreciation of HR and 0 was based on three different plants of the same genotype.

### Assessment of Ploidy and Genome Composition in SHs and Derived BCs

#### Determination of Ploidy Level by Classical Cytological Methods

##### Flow cytometry

The first pair of leaves of *in vitro* plants that were 8 weeks old were chopped with a razor blade in 800 μL of LB buffer, which was then filtered through a 25 μm mesh. Subsequently, 12.5 μL of buffer containing 1 μg/mg propidium iodide (PI) were added (Doležel et al., 2007). The nuclear DNA content was determined using a Becton Dickinson FacScan flow cytometer and the data analyzed using CellQuest software.

##### Determination of the ploidy level using cytological methods

Root tips of 1–2 cm in length were harvested from young plants grown *in vitro* on MS medium (Murashige and Skoog, 1962) containing 1 mg L^–1^ NAA, fixed in ethanol/acetic acid (3:1) and stored in 70% ethanol at 4°C. The root tips were washed twice in distilled water (10 min each), digested with a 1% Cellulase Onozuka R-10, 0.4% Cytohelicase and 0.4% Pectolyase in citrate buffer at pH = 4.8 (30 min) and squashed in 45% acetic acid. After the cover slips were removed in liquid nitrogen, slides were dehydrated by placing them sequentially in 70, 90, and finally 100% ethanol. Chromosomes were stained using 4′,6–diamidine-2′-phenylindolehydrochloride (DAPI) and washed with 2xSSC and distilled water. Probes were mounted in Vectashield (Vectashield, Vector Laboratories) to preserve the staining. For chromosome counts, at least five cells with well-spread metaphase chromosomes were used for each clone. Best spreads were photographed with a digital camera attached to an epifluorescent microscope (Olympus BX 60 with appropriate filter for DAPI). For all cytogenetic evaluations of the parental lines, the diploid *blb*41 and tetraploid cultivated potatoes were used as internal standards.

#### Determination of the Composition of the Genome Using mcGISH

A modified multicolour GISH protocol (Kruppa et al., 2013) was used to simultaneously visualize the chromosomes of *Solanum bulbocastanum* (2*n* = 2*x* = 24, A^*b*^A^*b*^) and *S. tuberosum* (2*n* = 4*x* = 48, AAAA). Therefore, total DNA was labeled with biotin-16-dUTP and digoxigenin-11-dUTP (Roche Diagnostics, Mannheim, Germany), respectively, using the random primed labeling protocol. The hybridization mixture contained 60 ng each of the labeled probes/slide, dissolved in a 15 μL mixture of 100% formamide, 20xSSC and 10% dextran-sulfate in the ratio of 5:1:4. In order to denature the probes DNA, the hybridization mixture was heated at 75°C for 8 min and then immediately put on ice. The chromosome spreads were denatured at 80°C in a mixture containing 100% formamide, 20xSSC and 10% dextran-sulfate in the ratio of 15:3:3. Hybridization was done at 42°C overnight. Streptavidin-FITC (Roche) and Anti-Digoxigenin-Rhodamine (Roche), respectively, were used in the detection phase. Counterstaining was performed using 2 μg/mL DAPI in Vectashield antifade mounting medium. The slides were screened using a Zeiss Axioskop-2 fluorescence microscope equipped with filters appropriate for DAPI, FITC, Rhodamine and the simultaneous detection of FITC and Rhodamine. Images were taken using a Spot CCD camera (Diagnostic Instruments) and processed using Image Pro Plus software (Media Cybernetics).

### Resistance to Late Blight

#### Detached Leaf Assay (DLA)

This assay used five leaves per clone and was replicated at least twice. The description of the *Pi* strain is given in section “Field Test.” One drop (1 μL) of a *Pi* suspension of 15 × 10^3^ zoosporangia/ml was placed on the underside of the leaves, which were flipped after 1 day and incubated at 16°C and 95% relative humidity at a light intensity of 2.1 μmol m^2^ s^–1^ and incubated for 5 days. After 5 days the size of the necrotic areas and mycelium development were estimated on a scale from 1 (no symptoms visible) to 9 (completely necrotic and covered with mycelium). The protocol for evaluating resistance using the DLA is described by Thieme et al. (2008). The *Pi* inoculum was maintained for 1 year on tuber slices of a highly susceptible variety without any R-genes (cv. ‘Adretta’) in order to maintain a high level of virulence.

#### Field Test

Field tests were carried out at the Julius Kühn-Institut (JKI) Federal Research Centre for Cultivated Plants Trial Station for Potato Research, Groß Lüsewitz 18190 Sanitz, Germany. Clones were cultivated in double-row plots with ten plants per plot. A strip of hemp 3 m in width was planted around the field to ensure wind protection and to maintain a humid environment. Irrigation was conducted in the early morning and in the evening if necessary. Control plots of varieties highly resistant (cv. ‘Sarpo Mira’) and susceptible (cv. ‘Adretta’) to late blight were also planted in the field. The inoculum of *Pi* consisted of a mixture of common races collected in the field in the year before and refreshed the following year with an inoculum collected from natural infections (Hammann et al., 2009). The virulence of the isolate was evaluated under field conditions using the SASA differential set carrying the *R* genes *R1* to *R11* (Black et al., 1953). It was found highly aggressive as it overcame the resistance genes *R1* to *R8* and *R10* and *R11*. Probably due to their later maturation, *R9* plants were less infected. A continuous monitoring of the composition of the clonal lineages of the pathogen was not possible throughout the test period, but analyses of samples from 2014 to 2017 identified EU_13 mating type A2 as the predominant genotype in local populations. The lowest leaves of each last plant in a row were inoculated with a 5 ml spore suspension (15 × 10^3^ zoosporangia/ml) in the evening. The area of the potato tops attacked was scored as a percentage twice a week until the plants were mature. This was based on the Area Under Disease Progress Curve (AUDPC) and Relative Area Under Disease Progress Curve (rAUDPC) (Hammann et al., 2009). As rAUDPC is strongly associated with the maturity of the plants, it was transformed into delta (Δ) rAUDPC. RAUDPC represents the percentage of infection and Δ-rAUDPC the quantitative maturity-corrected transformation of rAUDPC (Shaner and Finney, 1977; Bormann, 2003; Truberg et al., 2009). The maturity of the parents, SHs and progenies was evaluated in a field trial that was treated with fungicides using a scale from 1 (very early) to 9 (very late). Fifteen plants per clone were cultivated and agronomic characters other than maturity were also assessed.

### Evaluation of Flowering, Yield, Tuber Shape/Appearance and Tuber Processing Qualities

#### Assessment of Flowering, Yield, Tuber Shape and Appearance

The SHs and offspring were repeatedly cultivated in a greenhouse for 2–5 years depending on genotype and breeding step. In addition to the evaluation of their resistance to pathogens, morphological characteristics, such as, plant habitus, flowering and the fruit (berry) development were assessed. Crossbreeding experiments with pollen of different potato varieties were carried out. The number of flowers on a single greenhouse plant was assessed and expressed on a scale: + [low number (*n* ≤ 5)], ++ [moderate number (*n* = 6–20)] and +++ [high number of flowers (*n* > 21)]. Fertile SHs and BC clones were selected for further use.

Tuber appearance in terms of shape, eye depth, skin texture were assessed and the results are summarized in Table 4 [scale: + (poor), +++ (very good)].

**TABLE 4:** Assessment of the agronomic traits of BC progenies derived from *S. bulbocastanum*, GLKS 31741 (*blb*41) (+) cv. ‘Delikat’ hybrids grown in a field for 2 or 3 years, including flowering, tuber number, weight and starch content as well as processing qualities: Discolouration of raw tuber tissue, of cooked tuber flesh (average for 3 years); the BC clones with best yield, qualities for agronomic traits and processing are highlighted in green.

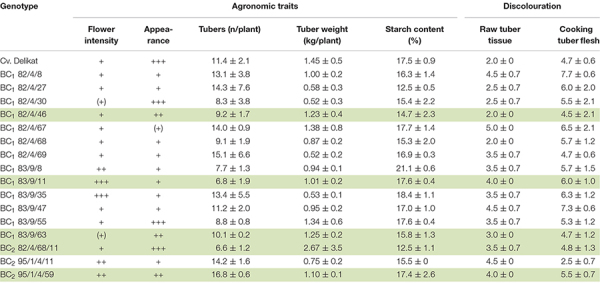

*Initial number, 15–20 plants per genotype and plot; tuber harvest per plot. Flower intensity on a scale: (+), +, low number (*n* ≤ 5); ++, moderate number (*n* = 6–20), and +++, high number of flowers (*n* > 21); Tuber appearance (including tuber shape, eye depth, skin texture) is summarized on a scale: (+), +, poor, +++, very good; Discolouration: 1 (best) – 9 (low quality).*

Yield (kg of tubers per plot), total number of tubers and tuber shape were assessed of plants that developed from the 15 tubers planted in each plot in the field. Yield of SHs and derived progenies were compared to that of the parental potato cultivar ‘Delikat’.

Processing quality was based on the discolouration of raw tuber tissue, flesh after cooking and the starch content.

#### Assessment of Discolouration of Raw Tuber Tissue

Four tubers per genotype were used 4–5 months after harvest. Using a cork-borer two tissue cylinders were cut out parallel to the long axis of each tuber. After 24 h, the discolouration was visually estimated in terms of intensity of gray to black on a scale from 1 (not discolored) to 9 (black). Cultivar ‘Delikat’ was used as a reference, as its reaction is well known.

#### Discolouration of Tuber Flesh After Cooking

Four medium-sized tubers per genotype were cooked, hot peeled, cut longitudinally and the cut surface placed facing down on a plate. Discolouration was estimated on a scale from 1 to 9 after 24 h and compared with the reference samples.

#### Starch Content

Starch content was determined, by calculating the difference between the weight of a tuber in air and in water using an under-water balance EORO-KUW-2000 (Fischer KG Bielefeld, Germany).

### Statistical Analysis

The free statistical software R (R Development Core Team, 2010) was used for statistical calculations. The function mean() was used to calculate means and the function sd() was used to calculate standard deviations.

## Results

### Identification of *Rpi-blb1*, *Rpi-blb3*, *R3a*, and *R3b* Genes in *S. bulbocastanum blb*41, SHs and Derived BC Progenies and Assessment of Resistance to Foliage Blight

**FIGURE 1:**
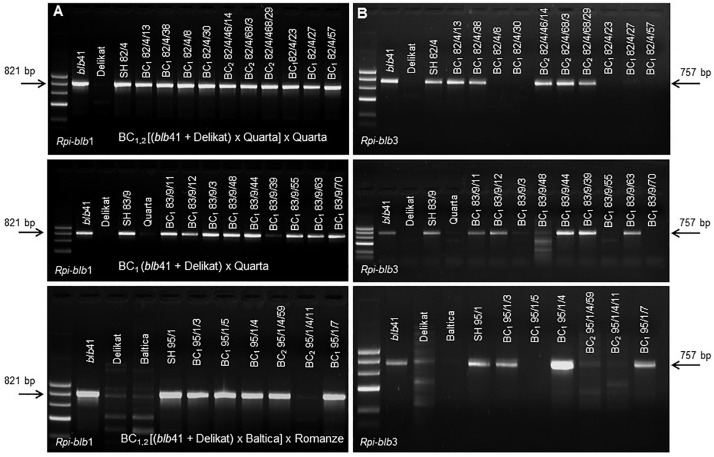
Molecular analysis using gene specific markers to determine the presence of **(A)** *Rpi-blb1* and **(B)** *Rpi-blb3* genes in selected somatic hybrids, their parental lines *S. bulbocastanum* GLKS 33741 (*blb*41), cv. ‘Delikat’ and different segregating BC_1_ progenies after back-crossing with cvs. ‘Quarta’ and ‘Baltica’ (from above to below, arrows indicate the target fragments).

Using specific markers it was possible to demonstrate the presence of the following resistance genes in *blb*41: *Rpi-blb1*, *Rpi-blb3, R3a*, and *R3b* and confirm their transfer into SHs and introgression into derived BC progenies (Figure 1, Tables 1–3, and Supplementary Figure S1). The fertile and best performing SH clones were further selected for ploidy (chromosome counts) and field assessments. All three SHs selected and the majority of their derived BCs carried both *Rpi* resistance genes (Tables 1–3). In one BC_1_ (83/9/39) gene specific markers indicated the presence of only the *Rpi-blb3* gene (Figure 1 and Table 2). This clone had a good score for resistance in the DLA (mean of 1.6), but a plus value for Δ-rAUDPC in the field (Table 2). The race-specific genes *R3a* and *R3b* were detected using gene specific markers in *blb*41 and the cultivars ‘Baltica’, ‘Quarta’ and ‘Sarpo Mira’, which were used as pollinators in back-crosses. Only *R3b* was detected in cv. ‘Romanze’ (Tables 1–3 and Supplementary Figure S1). The selected SHs 82/4, 83/9 and 95/1 carried the two durable resistance genes *Rpi-blb1* and *Rpi-blb3* and race-specific *R3a* and *R3b* genes (Tables 1–3). Hence, eight clones of the BCs derived from the three selected SHs and had specific fragments of all the resistance genes (Tables 1–3–highlighted in red, Figure 1. The three selected SHs and 30 of the 36 derived BC clones (listed in Tables 1–3), had a significantly high degree of *Pi* resistance in field trials, during the 1–4 years in which the intensity of the foliage blight attack varied (Tables 1–3 and Figures 3, 4A,B). In the first years, a large number of SHs was evaluated in the field (Figure 4A) and the BC clones of the best three SHs were evaluated in the years 2014 to 2016 (Figure 4B). The level of resistance varied from year to year and the susceptible cultivar ‘Delikat’ had a rAUDPC of only 50% in 2013. The maturity-corrected values of Δ-rAUDPC for these SH clones are lower than that of the susceptible standard cv. ‘Adretta’ and cv. ‘Delikat’, but rather similar to the wild species *blb*41, used as a control, which did not show any symptoms of foliage blight attack (rAUDPC = 0%) or cv. Sarpo Mira, used as resistant standard cultivar. The area under the disease progress curve (rAUDPC in %) and maturity data are shown in Figures 4A,B, and Δ-rAUDPC in Tables 1–3, for each SH family. Minus values of Δ-rAUDPC indicate a low level of susceptibility. Three of the SHs selected and eleven BC_1_–BC_2_ clones had all the genes and were only slightly susceptible with minus values of Δ-rAUDPC in the field (see Tables 1–3). Other BCs (18) with only one, two or three resistance genes had minus values for Δ-rAUDPC in the field trials in at least 1 year in Tables 1–3. The maturity of SHs and derived BC_1_ clones was intermediate between that of potato cultivar ‘Delikat’, with a maturity value of 5 and the very late maturing (value 9) wild species *blb*41 (Figures 4A,B). The majority of the BCs had values between 4.7 and 7.8, with some BC_1_ clones maturing later with values 8 and 9 (82/4/8, /27, /67 and 83/9/11, /35). Many of the BC_1_ clones had a low percentage rAUDPC with the last ones having low values in some years (Figure 4B). The SHs and their derived BC_1_ had minus values for Δ-rAUDPC when tested in the field, which was confirmed by the DLA results with one exception, clone 82/4/68/29, which had a good value in DLA (2.36 ± 0.84), but was susceptible in the field with a plus value of Δ-rAUDPC in 1 year (Table 1). It needs to be verified by more assays in the future.

### Agro-Infiltration With Effectors

**FIGURE 2:**
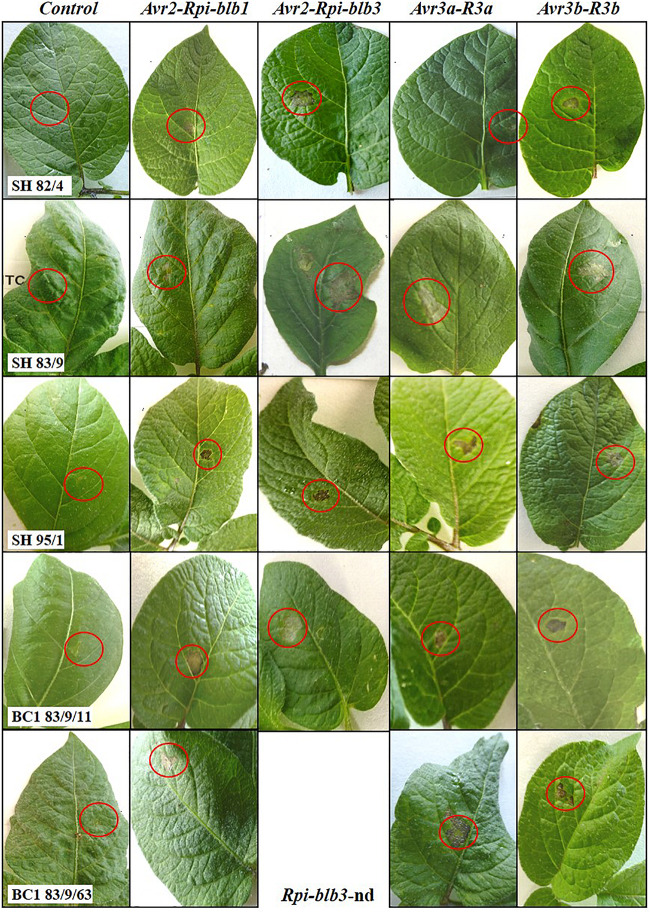
Hypersensitive reaction of leaves of *Solanum bulbocastanum* (GLKS 33741, *blb*41) (+) cv. ‘Delikat somatic hybrids (SHs) and selected back-cross progenies after agro-infiltration of *Avr-*effectors at 4*dpi*, which reveals the activity of corresponding resistance genes: *Rpi-blb1*, *Rpi-blb2*, *R3a*, or *R3b.* No visible reaction on leaves of SHs, BC_1_ by infiltration of the vector without effectors (empty vector control), nd-not determined.

The genes detected by gene specific markers after agro-infiltration with corresponding effectors were seen as elicitors of the hypersensitive response (HR) (Figure 2). Generally, after agro-infiltration these genes were associated with incompatible interactions and necrosis (Tables 1–3). There were also cases where genes were present but there was no hypersensitive reaction to the agro-infiltration of the *Avr* gene, as for example: BC_2_ 82/4/68/3 with the genes *Rpi-blb1* and *Rpi-blb3* but *Rpi-blb3* shows no HR (Table 1); or BC_1_ 83/9/8 with both *Rpi-blb3* and *R3a* genes showing no activity after agro-infiltration with *Avr2* and *Avr3a*; BC_1_ 83/9/55 and 83/9/70 in which the *R3a* gene, although present was inactive (Table 2). The eight BC clones with stacks of four genes that showed a HR are highlighted. Those are also best performing BCs in the DLA with constant minus values of Δ-rAUDPC in different years in the field after inoculation with *Pi* (Tables 1–3).

**FIGURE 3:**
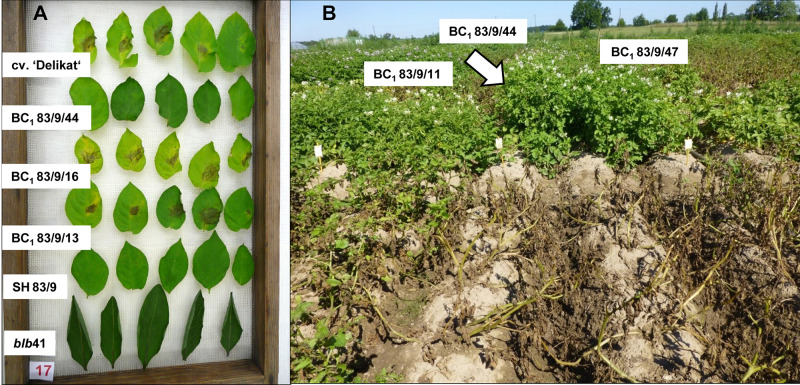
Evaluation of resistance to late blight in a greenhouse and the field **(A)** Detached leaf assay (DLA) showing resistant *S. bulbocastanum* GLKS 33741 (*blb*41), a resistant somatic hybrid, two susceptible and one resistant BC_1_ clones and the susceptible cv. ‘Delikat’ after infection with *Phytophthora infestans* (from below upward) **(B)** BC_1_ clones 83/9/44 (arrow), 83/9/11 (left) and 83/9/47 (right) derived from *blb*41 (+) cv. ‘Delikat’ somatic hybrid 83/9 in a field trial in 2015 expressed improved horizontal resistance to foliage blight.

**FIGURE 4:**
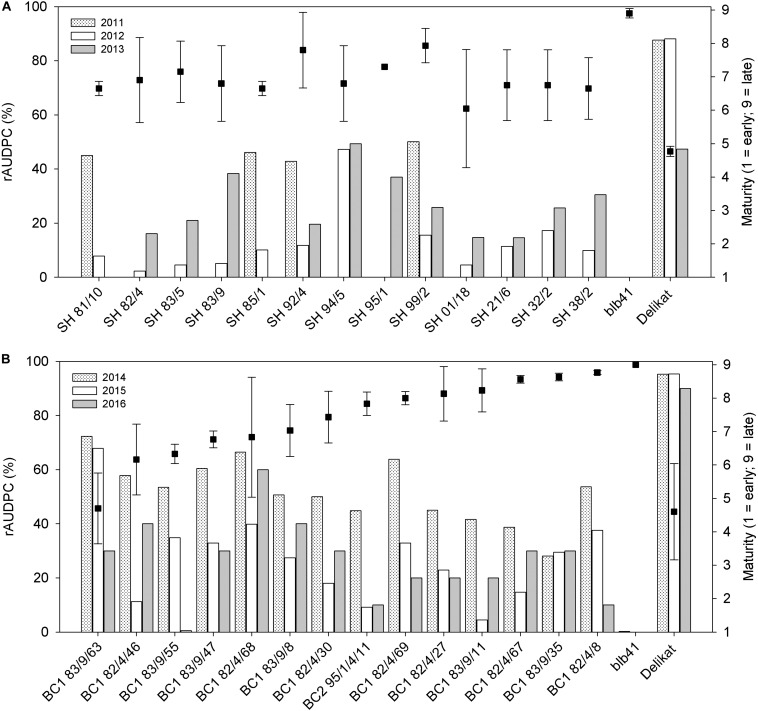
Percentage of foliage area infected by late blight expressed as relative Area Under Disease Progress Curve (rAUDPC) and maturity (1 = very early; 9 = very late), evaluated in the field, after artificial inoculation of parents, **(A)** Somatic hybrids and **(B)** Their back-crossed progenies, potato (+) *Solanum bulbocastanum* GLKS 33741 (*blb*41); **(A)** years: 2011, 2012, 2013; **(B)** Values for years 2014–2016).

### Ploidy and Genome Constitution of SHs and Derived BCs

**FIGURE 5:**
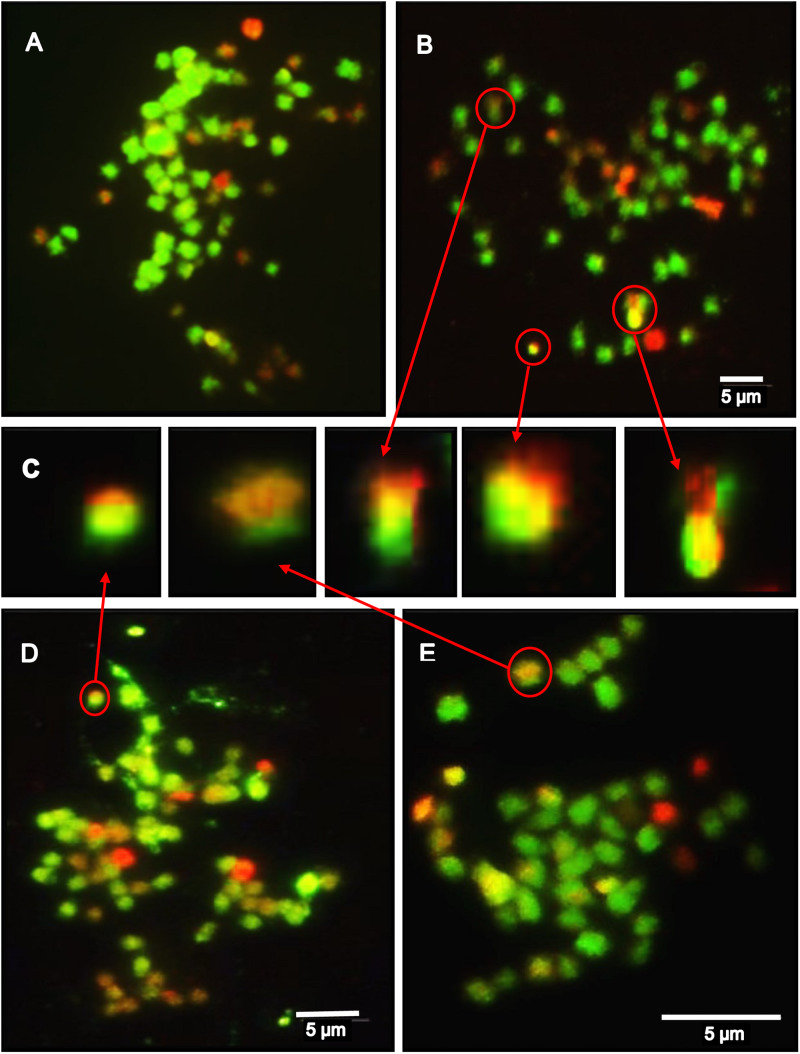
The results of a mcGISH analysis of the genetic composition of somatic hybrids (SH), back-cross progenies (BC_1_ and BC_2_) – examples; green labels *S. tuberosum*, red labels *S. bulbocastanum* GLKS 33741 (*blb*41) chromosomes: **(A)** SH 83/9 - total 72 chromosomes, in red *S. bulbocastanum* = 24; **(B)** BC_1_ 83/9/3 - total chromosomes 54, 10 red from *blb*41 **(C)** Detailed magnifications of the introgressions from *blb*41 (red) into potato chromosomes (green) in BC_1_ 83/9/3, SH 95/1 and BC_2_ 95/1/4/59 (as indicated by red arrows); **(D)** SH 95/1 – total 64 chromosomes with 24 *blb*41 chromosomes in red; **(E)** BC_2_ 95/1/4/59 (total 48 chromosomes, 4 red from *blb*41 and recombinations – with red and green); Yellow regions denote the complementarity of the respective sequences; bar = 5 μm.

As expected from previous investigations (Rakosy-Tican et al., 2015), the ploidy levels of the SH clones differed, one was hexaploid and two between pentaploid and hexaploid, i.e., aneuploids and asymmetric in their nuclear genetic constitutions (Supplementary Table S1 and Figures 5A–E). Our results indicate that after back-crossing with the following tetraploid cultivars: ‘Quarta’, ‘Baltica’, ‘Romanze’, and ‘Sarpo Mira’, all BC_1_ and BC_2_ clones have lower chromosome numbers (Supplementary Table S1). Nevertheless, mcGISH indicates that between 4 and 24 of the chromosomes were from the wild parent *blb*41 and the introgression of the DNA of wild species into potato chromosomes (Figure 5C).

### Yield of BC Clones in the Field and Processing Quality

**FIGURE 6:**
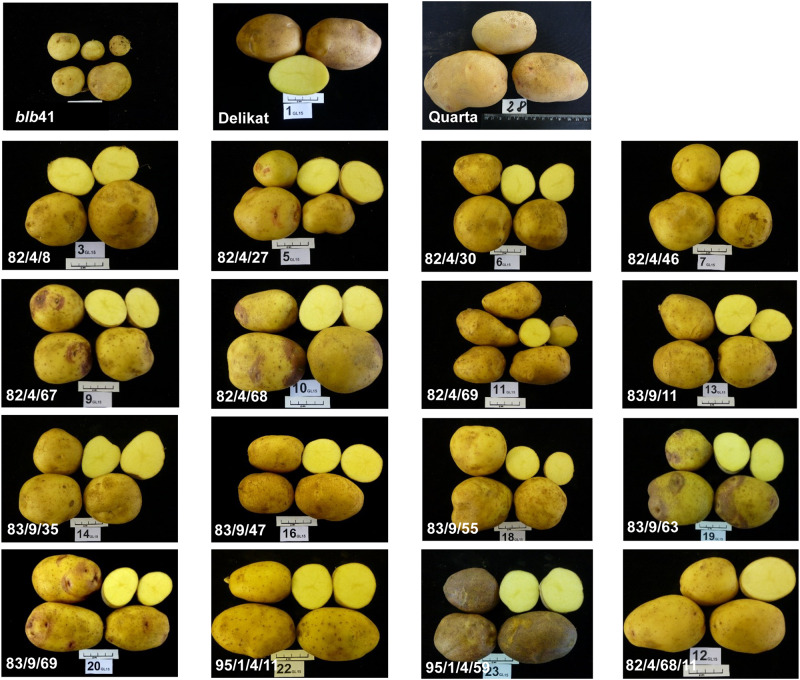
Size and appearance of tubers of parental lines and selected BC_1_ and BC_2_ (the last three below) progenies of *S. bulbocastanum* GLKS 31741 (*blb*41) (+) cv. ‘Delikat’ somatic hybrids after cultivation in a field.

**FIGURE 7:**
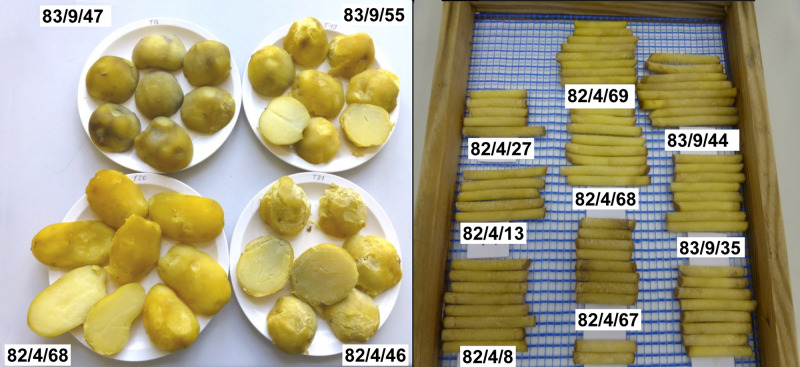
Assessment of quality traits: Discolouration of cooked tuber flesh (left) and raw tuber tissue of different BC progenies derived from *S. bulbocastanum* GLKS 31741 (*blb*41) (+) cv. ‘Delikat’ somatic hybrids 82/4 and 83/9 (back-crossed with cv. ‘Quarta’).

The BC clones grown in the field varied in terms of yield and shape of the tubers at harvest (Table 4 and Figure 6). The majority of the BC clones flowered and produced a satisfactory yield and number of tubers (Table 4 and Figure 6). Moreover, three BC clones (BC_1_ 82/4/30; BC_1_ 83/9/55; BC_2_ 82/4/68/11) produced tubers of good appearance and similar to those of cultivar ‘Delikat’ (Table 4 and Figure 6). The yield of two BC_1_ clones (82/4/67, 83/9/63) and one BC_2_ clone (95/1/4/59) was higher or similar to that of this cultivar, but the number of flowers and appearance of the tubers were low and acceptable, respectively. The starch content of the tubers of these clones varied from 12.5 to 21.1% and processing quality varied between scores 2.0 to 7.7 (Table 4). The BC_1_ clones 82/4/46; 82/4/68; 83/9/35; 83/9/55 produced tubers with good processing qualities (Figure 7). Useful pre-breeding clones are also highlighted in green (Table 4).

## Discussion

Somatic hybridization and the identification of resistance genes using gene-specific markers were used to transfer a stack of genes determining durable resistance to late blight (*Rpi-blb1* and *Rpi-blb3*) and the race-specific genes (*R3a* and *R3b*) into the potato cv. ‘Delikat’. This cultivar was shown to be a good candidate for somatic hybridization programs (Rakosy-Tican et al., 2015). By using *blb* accession GLKS 31741 (*blb*41), in which four R genes were identified and were introgressed into potato pre-breeding clones. Previously, it has not been possible to transfer this gene combination into potato either by classical breeding or by gene transfer.

The exploitation of the R genes for breeding research in potatoes has also been tested, in particular using genetic engineering approaches. Recently, more than 30 R genes found in species of wild potato resistant to late blight were mapped and 20 were cloned (Rodewald and Trognitz, 2013; Haverkort et al., 2016). These genes are available for the transfer into potato varieties and breeding lines. The company BASF developed the potato variety “Fortuna” derived from the cv. ‘Fontane’ by genetic transformation with two late blight R genes, *Rpi-blb1* and *Rpi-blb2* from *S. bulbocastanum* (Storck et al., 2012). This variety was not approved for the European market and it ceased to be cultivated. Zhu et al. (2012) have successfully stacked three other late blight resistance genes: *Rpi*-*sto1* (*S. stoloniferum*) homolog of *Rpi-blb1*, *Rpi*-*vnt1.1* (*S. venturii*) and *Rpi*-*blb3* (*S. bulbocastanum*). The susceptible cv. ‘Désirée’ became more resistant by introgressive hybridization and DLA indicated the presence of the stacked genes in the plants grown in a field over a period of 2 years (Zhu et al., 2012; Haesaert et al., 2015). In addition, two genes from *S. bulbocastanum* (*RB*, *Rpi-blb2*) and one R gene *Rpi-vnt1.1* from *S. venturii* were stacked using genetic transformation and transferred into the varieties preferred by farmers, ‘Désirée’ and ‘Victoria’, and transgenic clones of these plants were cultivated in Sub-Saharan Africa. These potato lines were completely resistant to late blight over three seasons in the field in Southwest Uganda (Gishlain et al., 2019). Currently, transgenic potato varieties are approved for cultivation in the United States, Canada and Bangladesh. In a 10 years research project on the durable resistance of potato to *Phytophthora* at Wageningen University and a Research Centre genetic engineering was used to produce potato plants resistant to late blight using R genes mainly from *Solanum bulbocastanum* (*blb*), *S. edinense, S. stoloniferum* (*sto*), *S. venturii* (*vnt*), and *S. chacoense* (*chc*). Four varieties were transformed with one to three R genes that originated from these wild potato species. The ‘Désirée’ clones containing stacks of either two R genes (*Rpi-blb3:sto1*; *Rpi-vnt1:chc1, Rpi-vnt1:sto1*) or three R genes (*Rpi-blb3:vnt1:sto1*) were not infected with *Pi* in field trials that lasted for 2 years (Haverkort et al., 2016). Recently, after using biotechnological improvement Simplot Plant Sciences has reported ‘Innate’ Gen 2 potatoes that are of high quality and show a strong resistance to late blight in the second generation^1^. Consequently, the use of biotechnology and MAS will increase the speed with which other genes conferring resistance can be incorporated into potato cultivars and their subsequent cultivation (Rietman, 2011; Chandel et al., 2015). Extensive investigations indicate that stacking of multiple R genes and monitoring how to deploy these stacks spatially and temporally could reduce fungicide use by over 80% (Haverkort et al., 2016).

Thus, genetic engineering could prove very useful if exempted from GMO rules in Europe. We used somatic hybridization, as the resulting plants are not genetically modified (directive 2001/18/EC–annex 1B)^2^. The use of somatic hybridization does enable to transfer new assortments of nuclear and cytoplasmic genes into regenerated plants and to overcome sexual incompatibility barriers (Rakosy-Tican et al., 2015; Thieme and Rakosy-Tican, 2017). Moreover, when asymmetric SHs are produced because of somatic incompatibilities (Rakosy-Tican et al., 2015), only a part of the nuclear DNA from the wild parent is present in the hybrids and derived BCs. In this study, mcGISH confirmed the introgression of *blb*41 DNA into BC clones, but more detailed analyses of chromosomes using FISH would identify the transferred resistance gene(s) and their location. Nevertheless, this study showed the introgression of DNA from wild species into potato cultivars using mcGISH.

The use of molecular markers and cytogenetic methods revealed that many of the SHs evaluated for resistance to *Pi* are asymmetric (Rakosy-Tican et al., 2015), which is confirmed in this study using indirect and direct cytogenetics and mcGISH. From the high number of the SHs between *blb*41 and five potato cultivars produced only those that had the basic potato characters in terms of morphology and fertility were selected (Rakosy-Tican et al., 2015) for assessing resistance to foliage blight and the important traits for a pre-breeding program. The DLA revealed that the three SH clones (*blb*41 + cv. ‘Delikat’) and BC_1_ derived progenies with both *Rpi-blb1* and *Rpi-blb3* genes showed little symptoms of infection with *Pi*. The analysis of the specific alleles at the *Rpi-blb1, Rpi-blb3, R3a*, and *R3b* loci indicate that in the following generations the resistance genes segregated. Presence of race-specific genes in the following BC generations was also caused by their presence in some of the cultivars used for back-crossing, like ‘Baltica’, ‘Sarpo Mira’, and ‘Quarta’ with *R3a* and *R3b* genes, and ‘Romanze’ with only the *R3b* gene. In a few cases, a lack of a direct correlation between the presence of one of the resistance genes and the level of resistance or functionality after agro-infiltration with the corresponding effector, indicate that the relevant genes were inactivated and the markers detected not only these resistance genes but also their analogs. Resistance is a complex trait with many effectors, epistatic or pleiotropic interactions (Lokossou et al., 2010; Aguilera-Galvez et al., 2018). It is known that in the susceptible haplotype (*rb*) there is a 18 bp deletion in the RB/*Rpi-blb1* gene (Bradeen et al., 2003). When the *Rpi-blb2* gene from *S. bulbocastanum* was transferred into potato cv. ‘Désirée’, there was not a positive correlation between gene expression in RT-qPCR assays and resistance to *Pi* in some cases (Orbegozo et al., 2016). Bearing in mind the genetic complexity of the SHs and BC clones (with many asymmetric nuclear constitutions in the SHs and a tendency for genetic stabilization in BC progenies), it is likely that the results of the RT-qPCR analysis will not always be correlated with the phenotypic resistance in DLA or field tests. Moreover, the evaluation of AUDPC in the field is also dependent on climate, which is the reason for presenting the data separately for each year in this study. The Δ-rAUDPC, however, confirmed the results of DLA in the field. It is likely that the tetrasomic inheritance in cultivated potato and the diverse nature of the genomic compositions of the SHs and derived progenies increased the complexity of the interactions, both genetically and between pathogen and host. Nevertheless, in the field trials SHs and four BC_1_/BC_2_ clones performed well in terms of tuber yield (Table 4) and resistance to foliage blight (Tables 1–3). For some of the hybrid clones the yield and number of tubers per plot was similar to those of the corresponding cultivar grown in the field trial, but tuber morphology in BC_2_ progenies need further improvement by breeding. The negative values of the Δ-rAUDPC of SHs and BC_1_ clones indicate that their resistance to foliage blight in the field assays was higher in at least 2 years. Compared to their parents more of the hybrids with both *Rpi-blb1* and *Rpi-blb3* genes survived an outbreak of late blight in the field in 2015 (data not shown). Moreover, the functionality of the genes identified by gene-specific markers was proven by agro-infiltration with corresponding *Avr*-effectors. The results demonstrate the functionality of stacked genes in the eight BCs, which were the most significant also in DLA and resistance in field assessments. The results demonstrate that the usage of somatic hybridization in combination with gene specific markers allow the transfer of multiple resistance traits (Thieme et al., 2008, 2010), which can also be tracked and stacked directly into potato cultivars. Incorporating more *R* genes into the potato might be the only way to increase the durability and level of resistance against late blight (Tan et al., 2010; Orbegozo et al., 2016). Other resistance mechanisms and traits can also be transferred along with those for which genes are already sequenced and, hence, can be easily tracked. Other authors (Davis et al., 2012) also report multiple resistances to late blight, viruses and aphids in the somatic fusion of potato and *S. bulbocastanum* and their crosses. In this study, tracking of these genes is not reported. Previous studies reported other resistant hybrids, which were fertile/infertile and either symmetric or asymmetric (Rakosy-Tican et al., 2015), which represent valuable material for further characterization and biotechnological improvement.

However, it is likely that *P. infestans* will eventually overcome the resistance to the newly released R gene variants. Therefore, various available strategies to control this insidious pathogen should be considered. It is necessary to deploy more cultivars that are both resistant and productive and our experiments showed that this is feasible. Moreover, some of our BC progenies of the three selected SHs are also good for producing chips and French Fries. Discolouration of the tissue of tubers, especially after cooking, and their starch content determine their table quality and value for the production of non-food products. Discolouration of the raw tuber flesh is caused by oxidation of phenolic compounds in the presence of polyphenol oxidase resulting in quinones, which is transformed into a dark pigment (Friedman, 1997). The level of phenolic compounds, activity of polyphenol oxidase and level of free amino acids also affect this process. Discolouration of tuber flesh after cooking is a key factor in determining their suitability for fresh consumption and processing (Wang-Pruski, 2007). The evaluation of the processing qualities of fusion hybrids and BC progenies will promote the exploitation of resistant clones as pre-breeding valuable genotypes. The results presented here underline the value of potato genotypes that are resistant and suitable for further processing.

In conclusion, this analysis of selected SHs and their BC_1_ and BC_2_ progenies has revealed eight progenies with a stack of four resistance genes, which are hypersensitive to corresponding *Avr*-effectors and resistant to late blight in DLA and the field (Δ-rAUDPC), the yield and processing qualities of some of which are good. These genotypes are valuable pre-breeding lines to produce potato cultivars with potentially durable resistance against foliage blight.

## Data Availability Statement

The raw data supporting the conclusions of this article will be made available by the authors, without undue reservation.

## Author Contributions

ER-T and RT developed the idea, produced the plant material, wrote the manuscript and contributed to the DLA tests, agro-infiltration, and data analysis. MN carried out the marker analysis. T-ED, KK, and MM-L did the cytogenetics and mcGISH analysis. TH and JK performed the resistance in the field, yield evaluations and functional profiling of resistance genes by agro-infiltration. All authors read and approved the final manuscript.

## Conflict of Interest

The authors declare that the research was conducted in the absence of any commercial or financial relationships that could be construed as a potential conflict of interest.

## Footnotes

1 http://www.innatepotatoes.com/gen-two

2 http://www.biosafety.be/PDF/2001_18.pdf

## Supplementary Material

FIGURE S1 An example of molecular analysis using gene specific markers to determine the presence of *R3a* and *R3b* genes in *S. bulbocastanum* GLKS 31741 (*blb*41), somatic hybrids *blb*41 (+) cv. ‘Delikat’ (SH) and their offspring (BC_1_); M: DNA Ladder 100 bp.

TABLE S1 Results of direct and indirect (flow cytometry) ploidy determination of selected somatic hybrids between potato cv. ‘Delikat’ and *Solanum bulbocastanum* (*blb*41) and their derived BC_1_ and BC_2_ progenies (nd-not determined).
