# Supplementary figures and images for: Introgression of Two Broad-Spectrum Late Blight Resistance Genes, Rpi-Blb1 and Rpi-Blb3, From Solanum bulbocastanum Dun Plus Race-Specific R Genes Into Potato Pre-breeding Lines
Source: Front Plant Sci. 2020 Jun 3;11:699. doi: 10.3389/fpls.2020.00699 (PMC7326066; doi:10.3389/fpls.2020.00699)

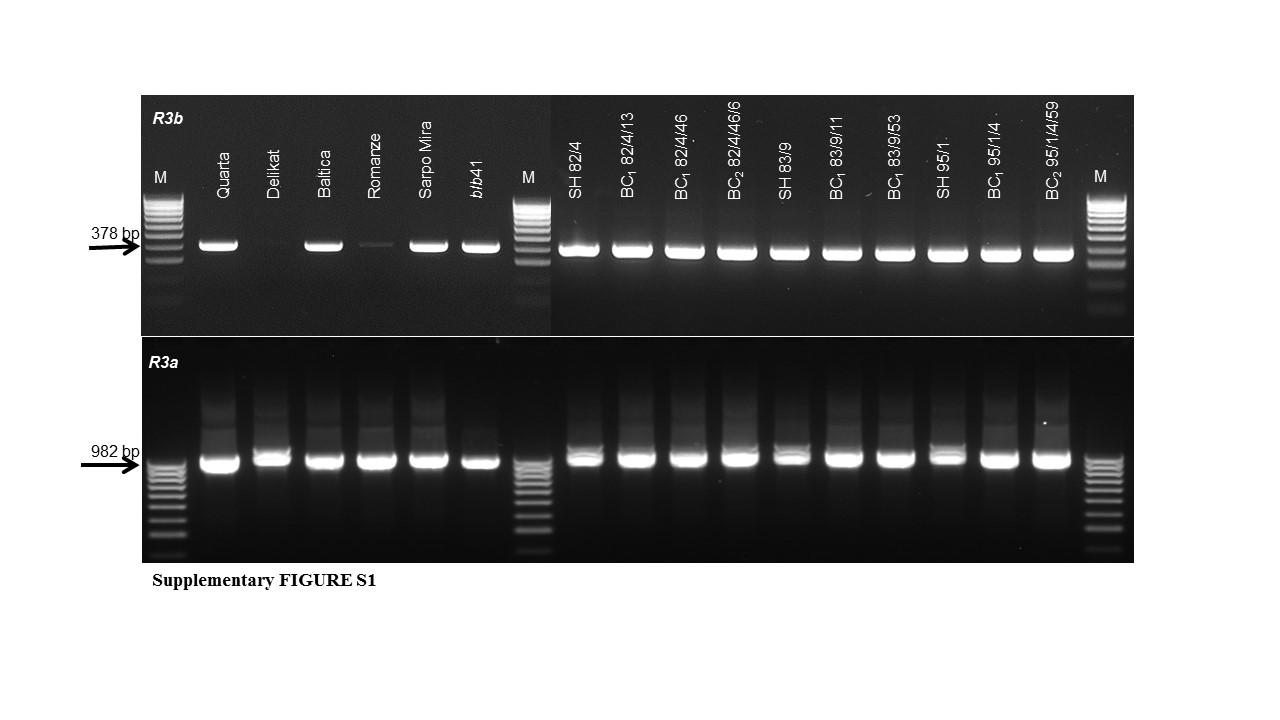

Supplement: FIGURE S1 — An example of molecular analysis using gene specific markers to determine the presence of R3a and R3b genes in S. bulbocastanum GLKS 31741 (blb41), somatic hybrids blb41 (+) cv. ‘Delikat’ (SH) and their offspring (BC1); M: DNA Ladder 100 bp. [file Image_1.JPEG]
