# Supplementary material for: Introgression of Two Broad-Spectrum Late Blight Resistance Genes, Rpi-Blb1 and Rpi-Blb3, From Solanum bulbocastanum Dun Plus Race-Specific R Genes Into Potato Pre-breeding Lines
Source: Front Plant Sci. 2020 Jun 3;11:699. doi: 10.3389/fpls.2020.00699 (PMC7326066; doi:10.3389/fpls.2020.00699)
Supplement: TABLE S1 — Results of direct and indirect (flow cytometry) ploidy determination of selected somatic hybrids between potato cv. ‘Delikat’ and Solanum bulbocastanum (blb41) and their derived BC1 and BC2 progenies (nd-not determined). [file Table_1.DOCX]

| **Genotypes** | **Ploidy - flow**  **cytometry (FC) - n** | | **Quantity of DNA (pg) - FC** | | **Chromosome counts**  **- DAPI staining** | |  |
| --- | --- | --- | --- | --- | --- | --- | --- |
| ***S. bulbocastanum*** | | 2 | | 1.14 | | 24 | |
| ***S. tuberosum*** | | 4 | | 2.73 | | 48 | |
| **SH 82/4** | | 5-6 | | 3.22 | | 48-60 | |
| **BC_1_ 82/4/4** | | 5-6 | | 2.94 | | 58 | |
| **BC_1_ 82/4/38** | | 4-5 | | 3.13 | | 54 | |
| **BC_1_ 82/4/68** | | 5-6 | | 3.34 | | 58-60 | |
| **BC_2_ 82/4/68/22** | | 5-6 | | 3.50 | | 60 | |
| **SH 83/9** | | 6 | | 3.99 | | 66-72 | |
| **BC_1_ 83/9/3** | | 4 | | 2.84 | | 54 | |
| **BC_1_ 83/9/27** | | 5-6 | | 3.37 | | 52 | |
| **BC_1_ 83/9/64** | | 5-6 | | 3.16 | | 52 | |
| **BC_1_ 83/9/63** | | 5-6 | | 3.16 | | 60 | |
| **SH 95/1** | | 5-6 | | 3.88 | | 62-64 | |
| **BC_1_ 95/1/3** | | nd | | nd | | 60 | |
| **BC_1_ 95/1/7** | | 4-5 | | 3.09 | | 58 | |
| **BC_2_ 95/1/4/11** | | 4 | | 2.86 | | 50 | |
| **BC_2_ 95/1/4/59** | | 4 | | 2.88 | | 50 | |

**Supplementary TABLE S1** Results of direct and indirect (flow cytometry) ploidy determination in the selected somatic hybrids (SH) between *Solanum bulbocastanum* (*blb*41) (+) *S.* *tuberosum* (cv. ‘Delikat’) and their derived BC_1_ and BC_2_ progenies (nd = not determined); Red somatic hybrids, green BC_1_ and yellow BC_2_ generations
